# Pathogenetic, Clinical, and Prognostic Features of Adult t(4;11)(q21;q23)/*MLL-AF4* Positive B-Cell Acute Lymphoblastic Leukemia

**DOI:** 10.1155/2011/621627

**Published:** 2011-11-15

**Authors:** F. Marchesi, K. Girardi, G. Avvisati

**Affiliations:** Unit of Hematology, Stem Cell Transplantation, Transfusion Medicine and Cellular Therapy, Campus Bio-Medico University Hospital, Via Àlvaro del Portillo 200, 00128 Rome, Italy

## Abstract

Translocation t(4;11)(q21;q23) leading to formation of *MLL-AF4* fusion gene is found in about 10% of newly diagnosed B-cell acute lymphoblastic leukemia (ALL) in adult patients. Patients expressing this chromosomal aberration present typical biological, immunophenotypic, and clinical features. This form of leukemia is universally recognized as high-risk leukemia and treatment intensification with allogeneic hematopoietic stem cell transplantation (HSCT) in first complete remission (CR) could be a valid option to improve prognosis, but data obtained from the literature are controversial. In this review, we briefly describe pathogenetic, clinical, and prognostic characteristics of adult t(4;11)(q21;q23)/*MLL-AF4* positive ALL and provide a review of the clinical outcome reported by the most important cooperative groups worldwide.

## 1. Introduction

The chromosomal translocation occurring between the band 21 of the long arm of chromosome 4 and band 23 of the long arm of chromosome 11 [t(4;11)(q21;q23)] and leading to the generation of the fusion gene *MLL-AF4* is one of the most recurrent chromosomal aberrations observed in acute lymphoblastic leukemia (ALL). However, a diagnosis of t(4;11)(q21;q23)/*MLL-AF4* positive ALL in adult patients is a rare event, considering the relative low incidence of ALL in adult population. In spite of its rarity, this form of leukemia is of clinical interest because it is universally recognized as a unique and separate biological entity with characteristic immunophenotypic and clinical features. Here, we briefly describe pathogenetic, clinical, and prognostic characteristics of adult t(4;11)(q21;q23)/*MLL-AF4* positive ALL and review the therapeutic approaches proposed for its treatment by most of the important cooperative groups worldwide.

## 2. Pathogenetic Aspects of MLL Rearrangements

Mixed-Lineage-Leukemia (*MLL*) gene is one of the most frequently involved genes in hematologic malignancies, in particular in some forms of acute leukemia, both lymphoblastic and myeloid; the *Atlas of Genetics Oncology* (http://atlasgeneticsoncology.org/Anomalies/11q23ID1030.html) reports 73 recurrent translocations and 54 chromosome loci as partner site of reciprocal translocations involving the band 23 of the long arm of chromosome 11 (11q23), in particular *MLL *gene. The *MLL* gene, located on 11q23, is the mammalian counterpart of *Drosophila trithorax* that plays an essential role in positive regulation of gene expression in early embryonic development and hematopoiesis (i.e., *Polycomb* and *Hox* genes) [1]. *MLL* encodes a 500 kD protein that contains multiple conserved functional domains including three AT hooks (near the N-terminal portion of MLL), four central zinc finger domains, and 210-aminoacid C-terminal SET domain. The last is responsible for its histone H3 lysine 4 (H3K4) methyltransferase activity which mediates chromatin modifications associated with epigenetic transcriptional activation [2]. MLL localization and stabilization depend on a proteolytic post-translational process activated by taspase1, a specialized protease cleaving the MLL protein into N-terminal 320 kD (MLL^n^) and C-terminal 180 kD (MLL^c^) fragments. These fragments are responsible for the transcriptional regulation of specific target genes, including many of the *HOX* genes, that are key regulators of normal and malignant hematopoiesis [3]. 

Several chromosomal aberrations can occur to the *MLL* gene, with two main action mechanisms: reciprocal translocations, resulting in in-frame fusion transcripts with various partner genes, and partial tandem duplication (PTD) of gene [4]. *MLL* gene translocations result in a chimeric fusion protein in which the N-terminal portion of the *MLL* gene is fused to the C-terminal portion of the gene fusion partners; the methyltransferase domain of MLL (SET domain) is invariably lost in MLL-fusion protein. These fusion genes may alter the normal cellular proliferation and differentiation processes, favoring leukemogenesis [5]. Several studies demonstrated that 11q23 is susceptible to double strand breaks resulting from inhibition of topoisomerase II [6]; this specific susceptibility can explain the high incidence of *MLL* aberrations occurring in secondary acute leukemias (i.e., therapy-related acute myeloid leukemia, especially after treatment with topoisomerase II inhibitors). Two distinct breakpoint cluster regions in the *MLL *gene could be distinguished: bcr1 and bcr2. Bcr1 encompasses approximately 3.5 kb from the start of intron 8 up to the first approximately 600 bp of intron 11, and bcr2 included approximately 200 bp immediately at the 5' boundary of exon 12. Ninety-five percent of breaks occurred within these 2 regions [7].

Recently published data have revealed 104 different MLL rearrangements of which 64 translocation partner genes are now characterized [8]. It is worth noting that all partner proteins are nuclear localization signals and play function as potent transcriptional factors. The most common fusion partner genes of MLL, reported in order of frequency, are AF4, AF9, ENL, AF10, AF6, ELL, and AF1P. Interestingly, distinct *MLL* fusion partners suggest a possible role in the tropism of the leukemia because certain partner proteins not only convert *MLL* to an oncogenic fusion protein but also direct the lineage susceptibility for transformation; *MLL-AF4* expressing leukemias are mainly diagnosed as pro-B ALL in both pediatric and adult patients, whereas fusion partners *AF9, AF6*, or *AF10* are common in myelomonocytic or monoblastic acute myeloid leukemia subtypes [9]. It is difficult to imagine how unrelated proteins create oncogenic MLL chimeras that transform haematopoietic cells by similar mechanism. Several studies suggest that MLL fusion partners interact with a complex of proteins, that stimulate the activity of RNA-polymerase II, leading to genes deregulation and transformation in leukemia [10, 11]. These proteins include PTEFb and DOTL1. PTEFb is a dimer of CDK9 and cyclin T1 that phosphorylates the C-terminal domain of RNA polymerase II (CTD) for efficient transcription elongation [12]. AF4, in association with ENL and AF9, stimulates activity of the RNA polymerase II (RNA pol II)-CTD kinase pTEFb and the histone methyltransferase DOT1L show that fusing the pTEFb-interacting domain of AF4 family members to MLL is necessary and sufficient for leukemic transformation, while DOT1L is not sufficient [13]. Other studies suggest that DOT1L methyltransferase activity is crucial for Hox gene deregulation and transformation seen in leukemias with MLL rearrangements. (Table 1) [10–14].

However, these experimental models recapitulate *MLL*-rearranged AML and the development of models about MLL-fusion mediated ALL has proven more difficult, so the exact mechanism by which the translocation t(4;11)(q21;q23) leads to leukemogenesis is incompletely characterized. 

An early favored hypothesis was that haploin sufficiency of the *MLL *locus combined with a dominant-negative effect of the oncogenic fusion gene could lead to the loss of key *MLL* functions [15]. For describing *MLL*-fusion-mediated ALL, several mouse models and molecular experimental systems have been so far engineered. However, the first engineered mouse models have resulted in myelodysplasia or mature B-cell lymphomas. In fact, Chen and collaborators in a murine *Mll-Af4* knock-in model observed the development of a mixed lymphoid/myeloid hyperplasia or mature B-cell lymphomas (after prolonged latency), suggesting that *Mll-Af4*-induced lymphoid/myeloid deregulation alone is insufficient to produce malignancy [16]. Using also invertor technology for performing a conditional expression of Mll-Af4 in lymphoid lineage in mice, Metzler et al. found the development of mature lymphoproliferative disease, demonstrating that the stem cell in which the MLL fusion protein is expressed is not an uncommitted progenitor and that MLL-AF4 influences the phenotype of the tumour when expressed within cells of the lymphoid lineage [17]. Further studies in murine systems suggested an active role for MLL partners in leukemogenesis, through a dysregulation of gene expression in leukemic cells. In fact, Krivtsov et al. using conditional Mll-Af4 knock-in mouse (in which the MLL-AF4 fusion product is expressed within the context of the endogenous *MLL *locus) observed that the expression of *Mll-Af4* in lymphoid cells leads to in vitro leukemic transformation. It was associated with an overexpression of some genes, as *HoxA9 *and *Meis1*, observed in ALL and caused by an high H3K4 methyltransferase activity. Since the methyltransferase domain of MLL is invariably lost in MLL-fusion proteins, including *MLL-AF4*, it was found that *MLL-AF4* recruits DOT1L to MLL target genes, and promotes methylation stimulating transcriptional elongation of genes that are normally primed but not fully transcribed [18]. Using a murine retroviral model, Faber et al. demonstrated that the suppression of *HoxA9 *causes apoptosis in cell expressing an *Mll*-fusion, suggesting that *Hox *genes are necessary for survival of leukemic cells [19].

In addition, using microarray technology, it was demonstrated that the MLL-rearrangement cells present an upregulation of HOX genes (HOXA9, HOXA10, and HOXC6, together with the MEIS1 HOX cofactor), emphasizing the central role of deregulation of this class of genes in the pathogenesis of MLL-rearrangement ALL [20]. Fernando and collaborators showed that HOX gene overexpression in B and T-lineage leukemias with MLL translocations, cause a block at an early stage of cell differentiation and an aberrantly increased cell survival [21]. How MLL gene rearrangement upregulates HOX genes is unknown, but two prevalent models have been established: transactivation and dimerization, that are not mutually exclusive. The activation of target genes by MLL fusions can also be mediated through histone modifications and methylation, suggesting a crucial role of epigenetic regulation in oncogenesis [22]. Several studies demonstrated that the dimerization of the MLL N-terminal portion is necessary for leukemogenic transformation, because it immortalizes hematopoietic cells and imposes a reversible block on differentiation; the analysis of transiently transfected cells showed that dimerization of the fusion protein activated transcription (nearly 250-fold) in a dose-dependent manner. In addition, this mechanism causes a resistance to cell degradation by specific cell-cycle ligase in MLL fusion protein [23]. Dimerization of MLL N-terminal portion of MLL gene converts it into a transcriptional transactivator, leading to upregulation of HOX proteins, especially *HOXA9* and *MEIS1*, that are overexpressed in a wide variety of some leukemias (T-ALLs, acute myeloid leukemia and biphenotypic leukemia) and that act, at least partially, through activation of the proto-oncogene *MYB* [24]. In general, *HOX* transcription factors are not only master controls of embryonic development but they also direct normal hematopoietic differentiation. *HOX *expression is high in stem cells and early precursors and needs to be downregulated for maturation. Therefore, a continuous ectopic *HOX *expression will block differentiation and create a rapidly proliferating preleukemic precursor pool. Obviously, other mechanisms are involved in the pathogenesis of MLL rearranged ALL, suggesting a crucial role of epigenetic modification of chromatin region connected with MLL translocation. For example, Gessner and collaborators showed a link between *MLL/AF4* and telomerase, a key element of both normal and malignant self-renewal. Moreover, they examined the influence of *MLL/AF4* on the expression of TERT (telomerase reverse transcriptase) coding for the telomerase protein subunit, and subsequently telomerase activity in t(4;11)-positive ALL, showing that *MLL/AF4* through the expression of a specific gene, such as HOXA7 unbalanced TERT expression and accelerated telomere shortening [25]. In addition, MLL-rearranged ALL is frequently associated with an overexpression of fms-related tyrosine kinase 3 (FLT3), that seems contributing, at least in part, to resistance to chemotherapy [26].

## 3. Clinical Features

### 3.1. Incidence

The incidence of t(4;11)(q21;q23)/*MLL-AF4* positive ALL has a characteristic bimodal age distribution with a major peak in early infancy, occurring in over 50% ALL cases in infants aged less than 6 months, in 10–20% of older infants, in about 2% of children, and in almost 10% of adults [27–29].

The presence of a translocation t(4;11)(q21;q23) or a fusion gene *MLL-AF4* is detected in almost 10% of newly diagnosed adult B-cell ALL and in about 30–40% of pro-B ALL subtypes [30–35]. 

In the international clinical trial of the Medical Research Council (MRC) UKALLXII and the Eastern Cooperative Oncology Group (ECOG) E2993, cytogenetic data from a total of 1522 adult patients with newly diagnosed ALL were centrally reviewed of 1003 cases in which cytogenetic analysis was successfully performed; 69 patients had a translocation involving the *MLL* gene located at 11q23 and the majority of these (*n* = 54) had a t(4;11)(q21;q23), with a global estimate incidence of 6.9% and 5.4% respectively [30].

### 3.2. Immunophenotype and Morphology

In the vast majority of t(4;11)(q21;q23)/*MLL-AF4* positive ALL, leukemic blasts have a typical antigenic profile, suggesting a postulated origin from the multipotent or very early CD10neg B-progenitor cells with a frequent coexpression of myeloid antigens: CD19, CD22, cyCD79a, HLA-DR, TdT, and CD34 are frequently and strongly expressed, CD24 and cyIgM are negative or weakly expressed, CD20 is rarely expressed whereas CD10 is always negative. CD15 and CD65 myeloid antigens are frequently expressed but CD13 and CD33 are negative. This immunophenotypic pattern can be also used to predict with relative precision the presence of a translocation between chromosome 4 and 11, and the typical and aberrant expression of some myeloid antigens can be useful for monitoring minimal residual disease (MRD) during the treatment, in order to establish need for treatment intensification [40, 41]. More studies have so far described the strong association between a CD10neg B-cell precursor immunophenotype (pro-B-cell ALL) and abnormalities of band 23 of chromosome 11, particularly in infant ALL but also in adult patients [34, 42]. 

Recently, it has been observed that the chondroitin sulfate proteoglycan neural-glial antigen 2 (NG2) is frequently expressed in ALL with *MLL* rearrangements and is relatively, though not absolutely, specific. In particular, Burmeister and collaborators showed NG2 expression in 184 newly diagnosed patients with CD10 negative B-cell ALL, studied in place of showed NG2 expression in 94% of *MLL-AF4* positive patients, in 87% of patients with other *MLL* rearrangements and only in 15% of *MLL* negative patients, suggesting the relative specificity of this marker in predicting aberration of the *MLL* gene [7, 43]. Concerning morphology features, no specific morphologic pattern is associated to the t(4;11)(q21;q23) ALL, but many cases are diagnosed as L2 French-American-British (FAB) subtype.

### 3.3. Clinical Presentation

The presence of t(4;11)(q21;q23) with expression of the fusion gene *MLL-AF4* characterizes a subset of ALL with aggressive clinical features. These patients at diagnosis frequently have an elevated white blood count (WBC), massive hepatosplenomegaly or lymphadenomegaly, higher LDH values, and frequent Central Nervous System (CNS) involvement, with a poor clinical outcome both in infants and in adults [29, 44]. In contrast to other forms of ALL, these patients are characterized by a frequent presence of disseminated intravascular coagulation (DIC) at diagnosis: Vey and collaborators have described 14 cases of DIC in 34 patients with t(4;11)(q21;q23)/*MLL-AF4* positive ALL at diagnosis (41%), a percentage significantly higher compared to other patients enrolled in the LALA-94 cooperative study of the France-Belgium Group for Lymphoblastic Acute Leukemia in Adults. In this study however, patients with this chromosomal aberration had a similar incidence of organomegaly and of CNS involvement compared with other forms of ALL [38].

The clinical trials 03/87 and 03/89 of the German Multicenter study group for treatment of adult Acute Lymphoblastic Leukemia (GMALL) have shown that patients with t(4;11)(q21;q23) ALL at diagnosis had a higher median WBC count (168.3 × 10^9^/L), a male predominance, increased CD65 expression, a younger age predominance, and a lower incidence of initial infections than other cytogenetic subgroups of pro-B ALL. However, no differences were observed for the presence of hepatosplenomegaly, initial bleeding, haemoglobin level at diagnosis, and also for the prevalence of CNS involvement [33]. More recent data from the same cooperative study group confirmed only some of these findings. In 184 adult patients with pro-B CD10neg ALL enrolled in two consecutive clinical trials (GMALL 6/99 and 7/03), *MLL-AF4* positive patients were characterized by a more aggressive clinical presentation, with higher WBC at diagnosis (median: 141 × 10^9^/L), but no difference was reported for age at presentation between* MLL/AF4* positive and negative patients [7]. In the Gruppo Italiano Malattie EMatologiche dell'Adulto (GIMEMA) 0496 protocol, 24 patients presented at diagnosis a t(4;11)(q21;q23)/*MLL-AF4* positive ALL. These patients had a median age of 39 years with no sex prevalence, more than 50% had at diagnosis a WBC number higher than 50 × 10^9^/L; all patients presented a B-cell phenotype and none of these patients were characterized by the expression of CD13 and CD33 myeloid antigens compared to the other cytogenetic-molecular subgroups [31]. Recently Cimino and collaborators describe the largest cohort of patients with *MLL-AF4* positive ALL, analyzing the clinical course of 46 adult patients enrolled into 2 successive multicenter clinical trials (GIMEMA 0496 and LAL 2000): all cases presented a pro-B immunophenotypic pattern; the median age of patients was 39 years without sex predominance, WBC count at diagnosis was lower respect to previously described series with a median value of 60 × 10^9^/L and the median haemoglobin value and platelet counts were 9 g/dL and 33 × 10^9^/L, respectively [45].

### 3.4. Clinical Outcome and Prognostic Considerations

Cytogenetic and molecular analysis of leukemic cells at diagnosis are cornerstones for the prognostic stratification of ALL patients at onset of disease because they are independent factors in predicting clinical outcome of patients. Stratification of ALL patients according to cytogenetic and molecular characterization helps establish the best postremission therapy for individual patients, including the possibility of consolidation treatment intensification and allogeneic hematopoietic stem cell transplantation (HSCT).

The presence of 11q23 chromosomal aberrations with alteration of *MLL* gene is generally recognized as an unfavourable prognostic characteristic of some forms of ALL. [46–49]. A t(4;11)(q21;q23)/*MLL-AF4* positive ALL is generally considered as a high risk leukemia, characterized by a poor clinical outcome respect to other cytogenetic risk groups. Based on more recently published data, adult ALL patients can be separated in three different prognostic groups according to specific cytogenetic and molecular aberrations found at onset of disease: a standard, an intermediate, and an high-risk group, including t(4;11)(q21;q23)/*MLL-AF4* positive patients (Table 2). Moreover, in several studies it has been demonstrated that cytogenetic-molecular risk and WBC count at diagnosis were the main prognostic factor influencing DFS and OS in adult ALL patients. However, despite the great relevance of cytogenetic and molecular aberrations on clinical outcome of the adult ALL patients, a correct risk stratification useful to modulate the intensity of treatment needs to be integrated with other clinical baseline data and with important dynamic parameters, such as the timing of reaching morphologic complete remission (CR) and the quantification of MRD after induction or consolidation therapy using immunophenotypic or molecular methods (Table 3).

Table 4 summarizes the most relevant studies performed by cooperative multicenter groups worldwide in adult ALL, showing clinical outcome of patients t(4;11)(q21;q23)/*MLL-AF4* positive. Due to the relatively low incidence of this chromosomal aberration, not all these studies show specific data about t(4;11)(q21;q23)/*MLL-AF4* positive patients, but focus more generally on non-Philadelphia-positive high-risk patients (i.e., age >30 years, WBC count >30×10^9^/L, t(4;11)(q21;q23)/*MLL-AF4* positive patients, t(1;19)/E2A-PBX1 positive patients, low hypodiploidy karyotype, CNS involvement), considering the similar clinical outcome observed for this heterogeneous group of patients. Globally considered, these studies all suggest that patients with t(4;11)(q21;q23)/*MLL-AF4* positive ALL have a poor clinical outcome compared to others non-high-risk patients and are potential candidates for postinduction intensification with HSCT in presence of an HLA-matched family donor. However, contrasting data about the best therapeutic approach of this ALL subtype have been reported. Results from MRC UKALLXII/ECOG 2993 trial [30] showed that t(4;11) alteration still identified a cohort of patients with poor clinical outcome, despite the treatment intensification with HSCT in first CR, due in part to high incidence of relapse after HSCT and in part to deaths in CR for complications related to transplant. By contrast, data from the LALA-94 study suggest that postinduction intensification with HSCT in t(4;11)/*MLL-AF4* positive patients was associated with a significantly improved DFS with respect to others patients and that this therapeutic strategy results in a similar clinical outcome in terms of DFS and OS in both standard and non Philadelphia-positive high-risk patients [38]. The advantage of HSCT in this setting of patients has been demonstrated in GMALL 04/89 study; indeed, in this study in which HSCT was planned in first CR as intensification after consolidation treatment, no differences in terms of both probability of OS and probability of continuous complete remission (CCR) have been observed between *MLL-AF4* positive and negative adult ALL patients [33].

Considering all these studies, due to limited number of adult patients with this chromosomal aberration, it is not possible at present to definitively establish the real role of intensification treatment with HSCT. In general, the low occurrence of leukemia relapse in patients undergoing HSCT could be a indirect evidence of advantage of this treatment strategy, but the high incidence of fatal complications transplant-related may be responsible of the lack of OS improvement. Moreover, definitive results of MRC UKALLXII/ECOG E2993 trial have shown interesting results about the role of HSCT in ALL. The donor versus no donor analysis demonstrated a statistically significant 5-year OS improvement only in standard risk adult patients undergoing intensification with HSCT in first CR but not in high-risk patients, in which an increase in transplant-related mortality was observed [50]. These findings are potentially able to change the universally accepted idea about the HSCT role as intensification treatment in high-risk ALL patients. Unfortunately, no subanalysis on cytogenetic risk stratification was performed in this study, because patients at diagnosis were not stratified according to cytogenetic results with the exclusion of Philadelphia-chromosome positive patients. 

However, also considering the similar results recently obtained in Philadelphia-negative ALL patients undergoing HSCT both from an HLA-matched family and from a high-quality matched unrelated donor [51], the European Group for Blood and Marrow Transplantation (EBMT) guidelines consider HSCT from a sibling donor or from a well-matched unrelated donor as a standard of care in adult patients with high-risk ALL [52].

The decision to intensify consolidation treatment in ALL can be facilitated by the MRD assessment in ALL sub-types expressing specific chromosome aberrations leading to formation of fusion genes. In particular, the first recognized method monitoring MRD was the detection of fusion gene levels expression by Polymerase Chain Reaction (PCR) [53]. So far, several studies have supported the role of MRD monitoring using molecular PCR-based methods; all these studies show a high percentage of leukemia relapse in patients with a result above 10^−4^ or 0.01% MRD after induction or consolidation therapy [35, 54, 55]. As for MRD monitoring using specific probes for *MLL-AF4*, there are only limited published observations; in a prospective study of the GIMEMA group, MRD positivity after consolidation therapy in about 25 consecutive patients with t(4;11)/*MLL-AF4* positive ALL, significantly correlates with a higher cumulative incidence of leukemia relapse and an inferior OS. Moreover, all patients with a persistent or reconverted PCR-positivity status after consolidation subsequently experienced a hematologic relapse of the disease [56].

## 4. Conclusions

t(4;11)(q21;q23)/*MLL-AF4* positive adult ALL remains an attractive leukemic subtype because of special pathogenetic and clinical aspects with respect to the other ALL forms. Prognosis of this form of ALL in adults patients remains poor despite several ongoing clinical and biological studies to improve clinical outcome. One of the most important questions in this setting remains the role of HSCT as consolidation treatment in first CR; even though this approach is the most used [52, 57], data obtained by international cooperative groups worldwide are controversial. 

In our opinion, HSCT from an HLA-matched family or high-quality unrelated donor remains a valid strategy for treatment intensification in first CR but this procedure, considering the high incidence of transplant-related mortality, should be performed primarily in those patients who have molecular MRD positivity after consolidation therapy. Moreover, a possible strategy to improve the clinical outcome of these patients could be the use of a more effective induction-consolidation therapy with the aim of reaching the molecular MRD negativity after consolidation therapy. To achieve this goal, the use of more aggressive pediatric-like regimens with higher dose of nonmyeloablative drugs can be an option. In the absence of specific recommendations and considering all the published studies, it is our opinion that, in patients with a negative MRD, considering the relatively low risk of leukemia relapse, a treatment intensification with HSCT should be performed only in case of reappearance of a PCR-positivity during the maintenance treatment or during the follow-up, even in absence of a clear hematologic relapse, as is currently recommended for acute promyelocytic leukemia.

## Figures and Tables

**Table 1:** The most common fusion partner genes of *MLL*: locations and functions.

Partner gene	Location	Function
*AF4*	4q21 Nuclear	Leads to RNApol-II activation and to transcriptional elongation
*AF9*	9p22 Nuclear	In association with ENL, DOT1L, and AF4, activator of RNApol-II kinase p-TEFb
*ENL*	19p13.3 Nuclear	Elongation factor. In association with AF9, DOT1L and AF4 activator of RNApol-II kinase p-TEFb
*AF10*	10p12 Nuclear	Transcriptional factor
*ELL*	19p13.1 Nuclear	Elongation factor interacts with a nuclear protein related to AF4
*AF6*	6q27 Cytoplasmatic	Multi-domain protein involved in signaling and organization of cell junctions during embryogenesis
*AF1P*	1p32 Cytoplasmatic	Part of the EGFR pathway, involved in receptor-mediated endocytosis of EGF

p-TEFb: positive transcription elongation factor b. p-TEFb phosphorylates serine residues of the carboxy-terminal domain of RNApol-II; RNApol-II: RNA polymerase II; CTD: carboxy-terminal domain kinase; DOT1L: DOT1-like, histone H3 methyltransferase; EGFR: epidermal growth factor receptor; EGF: epidermal growth factor.

**Table 2:** Cytogenetic molecular classification of adult ALL based on more recently published data.

Risk group	Chromosomal/molecular aberrations	5y-DFS	5y-OS
STANDARD-RISK	Isolated 9p/p15-p16 deletions High hyperdiploidy Normal karyotype/no molecular aberrations	35–68%	48–80%

INTERMEDIATE-RISK	del(6q) Trisomy of chromosome 21 Trisomy of chromosome 8 t(1;19)/*E2A-PBX *	37–51%	35–40%

HIGH-RISK	t(9;22)/*BCR-ABL * t(4;11)/*MLL-AF4 * 11q23 MLL rearrangements Monosomy of chromosome 7 Low hypodiploidy/near triploidy Complex karyotype High *BAALC* expression Aberrations of *IKZF1* gene	10–52%	15–35%

CR: complete remission; 5y-DFS: 5 years disease-free survival; 5y-OS: 5 years overall survival.

**Table 3:** Risk stratification in adult ALL (adapted from [36] and [37]).

Parameter	Favourable	Unfavourable
Age (years)	18–35	>35
WBC count	<30 × 10^9^/*L*	>30 × 10^9^/*L* (B-cell) >100 × 10^9^/*L* (T-cell)
Immunophenotype	Thymic	Pro-T, Mature T Pro-B CD20 expression
Cytogenetic/molecular data	del(9p) High hyperdiploidy	t(9;22)/*BCR-ABL * t(4;11)/*MLL-AF4 * Low hypodiploidy Complex karyotype High *BAALC* expression Aberrations of *IKZF1* gene
Time to CR	Early	Late (>3-4 weeks)
MRD after induction therapy	Negative (<10^−4^)	Positive (>10^−4^)

WBC: white blood count; CR: complete remission; MRD: minimal residual disease; *BAALC*: brain and acute leukemia cytoplasmic gene; *IKZF1*: IKAROS family zinc finger 1 gene.

**Table 4:** Clinical outcome of t(4;11)(q21;q23)/*MLL-AF4* positive ALL in different cooperative trials worldwide.

Study	Year	No. Patients (Age)	% of patients t(4;11)/*MLL-AF4* positive	Treatment strategy	% of patients undergoing HSCT intensification	Global outcome (OS and DFS)	t(4;11)/*MLL-AF4* positive ALL outcome
MRC UKALLXII/ECOG E2993 [30]	2007	1522 (15–65)	5.4%	Intensification with HSCT for Ph+ and for patients younger than 50 years with HLA-matched family donor	21%	5y-DFS: 38%(^a^) 5y-OS: 43%(^a^)	5y-DFS: 24% 5y-OS: 24%
GIMEMA LAL0496 [32]	2003	403 (15–60)	6%	Intensification with HSCT only for Ph+ patients	20%	5y-DFS: 31% 5y-OS: 31%	5y-CCR: 15% 5y-OS: 23%
GMALL 04/87–89 [33]	1998	611 (15–65)	3.6%	Intensification with HSCT for younger high risk patients with HLA-matched family donor	na	5y-CCR: 45% 5y-OS: 40%	5y-CCR: 40% 5y-OS: 41%
NILG-ALL 09/00 [35]	2009	280(16–65)	7.3%	Intensification with HSCT in patients MRD+ after consolidation	31%	5y-OS: 34%	5y-OS 27%(^b^)
LALA94 [38]	2006	922 (15–55)	6%	Intensification with HSCT in high risk and CNS+ patients	19%	5y-DFS: 30% 5y-OS: 33%	5y-DFS: 30% 5y-OS: 38%
GRAALL-2003 [34]	2009	225 (15–60)	9.5%	All patients were Ph negative. Intensification with HSCT in high risk patients	31%	3.5y-DFS: 55% 3.5y-OS: 60%	3.5y-DFS: 52%(^b^) —
PETHEMA ALL-93 [39]	2005	222 (15–50)	4%	Intensification with HSCT for patients with HLA-matched family donor	31%	5y-DFS: 35% 5y-OS: 34%	Same results

(^a^)data relative at Ph negative patients; (^b^)data relative at clinical outcome of all non-Ph+ high-risk patients evaluated, including t(4;11)/*MLL-AF4* positive patients. OS: overall survival; DFS: disease-free survival; CCR: survival in continuous complete remission; CR: complete remission; HSCT: allogeneic hematopoietic stem cell transplantation; Ph+: Philadelphia-positive patients; MRD: minimal residual disease; CNS: central nervous system; MRC: British Medical Research Council; ECOG: Eastern Cooperative Oncology Group; GIMEMA: Gruppo Italiano Malattie EMatologiche dell'Adulto; GMALL: German Multicenter study group for treatment of adult Acute Lymphoblastic Leukemia; NILG: Northern Italy Leukemia Group; LALA: France-Belgium Group for Lymphoblastic Acute Leukemia in Adults; GRAALL: Group for Research on Adult Acute Lymphoblastic Leukemia (including the former France-Belgium Group for Lymphoblastic Acute Leukemia in Adults, the French Western-Eastern Group for Lymphoblastic Acute Leukemia, and the Swiss Group for Clinical Cancer Research); PETHEMA: Programa para el Estudio del la Terapeutica en Hemopatía Maligna; na: not available.
